# 1694. Joint Fluid Cell Count of Septic Arthritis Patients Diagnosed with Positive Culture in Children

**DOI:** 10.1093/ofid/ofad500.1527

**Published:** 2023-11-27

**Authors:** Yuto Otsubo, Meiwa Shibata, Norikazu Ota, Hiroshi Hataya, Yuho Horikoshi

**Affiliations:** Division of Infectious Diseases, Department of Pediatrics, Tokyo Metropolitan Children’s Medical Center, Tokyo, Japan., Fuchu City, Tokyo, Japan; Division of Infectious Diseases, Department of Pediatrics, Tokyo Metropolitan Children's Medical Center, Fuchu, Tokyo, Japan; Division of Orthopedics, Tokyo Metropolitan Children's Medical Center, Tokyo, Japan., Fuchu City, Tokyo, Japan; Department of General Pediatrics, Tokyo Metropolitan Children’s Medical Center, Tokyo, Japan., Fuchu City, Tokyo, Japan; Division of Infectious Diseases, Department of Pediatrics, Tokyo Metropolitan Children's Medical Center, Fuchu, Tokyo, Japan

## Abstract

**Background:**

Septic arthritis is one of serious pediatric infections that could result motor comorbidities. Historically, joint fluid cell count of 50,000 and over cells/mm^3^ has been used for presumptive diagnosis of septic arthritis while awaiting for culture result. Our aim of study was to evaluate joint fluid cell count for diagnosis among septic arthritis patients confirmed with positive culture in children.

**Methods:**

Patients with septic arthritis were included at Tokyo Metropolitan Children's Medical Center from March 2010 to March 2023. Septic arthritis was confirmed with positive joint fluid culture for a pathogenic organism. Patients with negative joint fluid culture and patients without joint fluid cell count were excluded. Electrical charts were retrospectively reviewed for demographic data, timing of arthrocentesis, culture result and joint fluid cell count.

**Results:**

A total of 95 patients with septic arthritis were identified. After excluding patients with negative culture and without joint fluid cell count, 22 patients were included for our study. Median age was 5 (IQR: 2-10) years old. Girl ratio was 45%. The median joint fluid cell count was 19,575 (IQR: 6,806-47,388) cells/mm^3^. Patients with 50,000 and over cells/mm^3^ was 23%. The median time from onset to arthrocentesis was 3 (IQR: 2-5) days. The isolated organisms were methicillin-susceptible *Staphylococcus aureus* (50%), methicillin-resistant *S. aureus* (9%) and *Streptococcus pyogenes* (27%), *Streptococcus pneumoniae* (5%), *Klebsiella pneumoniae* (5%) and *Salmonella* spp (5%). The infected joints were hip (59%), knee (23%), ankle (14%), elbow (5%) and shoulder (5%). One patient had hip and ankle arthritis. Nine patients were complicated with septic osteomyelitis. The Pearson correlation coefficient between days from onset of symptoms to puncture and the number of joint cells was 0.093 (p=0.681). (Figure 1)

**Conclusion:**

Majority of patients with septic arthritis confirmed with joint fluid culture did not exceed joint fluid cell 50,000 cells/mm^3^.

**Disclosures:**

**All Authors**: No reported disclosures

